# A Novel 1726 Nm Laser Therapy for Durable Clearance of Seborrheic Dermatitis

**DOI:** 10.1111/jocd.70735

**Published:** 2026-02-15

**Authors:** Ali Al‐Mamoori, Vaughan Daniel, Sajjad Ghanim Al‐Badri, Ibrahim Khalil, Wael Al‐Daraji

**Affiliations:** ^1^ Department of Dermatology, Baghdad Teaching Hospital Baghdad Iraq; ^2^ Manchester University NHS Foundation Trust Manchester UK; ^3^ College of Medicine University of Warith Al‐Anbiyaa Karbala Iraq; ^4^ College of Medicine University of Baghdad Baghdad Iraq; ^5^ Dhaka Medical College and Hospital Dhaka Bangladesh; ^6^ Dermatology Department Ain Shams University Hospital Cairo Egypt

**Keywords:** 1726 nm laser, photothermolysis, sebaceous gland, seborrheic dermatitis

Dear Editor,

A 32‐year‐old male presented with a 5‐year history of refractory seborrheic dermatitis (SD) of the scalp, characterized by recurrent erythema, greasy scaling, and pruritus, worsened by stress and seasonal variation. Previous treatments, including ketoconazole shampoo, low‐potency topical corticosteroids, and salicylic acid shampoos, provided only transient improvement. The patient had no known immunodeficiency, systemic disease, or use of immunosuppressive medications.

Dermatological examination revealed confluent erythematous plaques with thick yellowish scale involving the vertex and frontal scalp (Figure 1A). Other seborrheic areas were clinically uninvolved.

**FIGURE 1:**
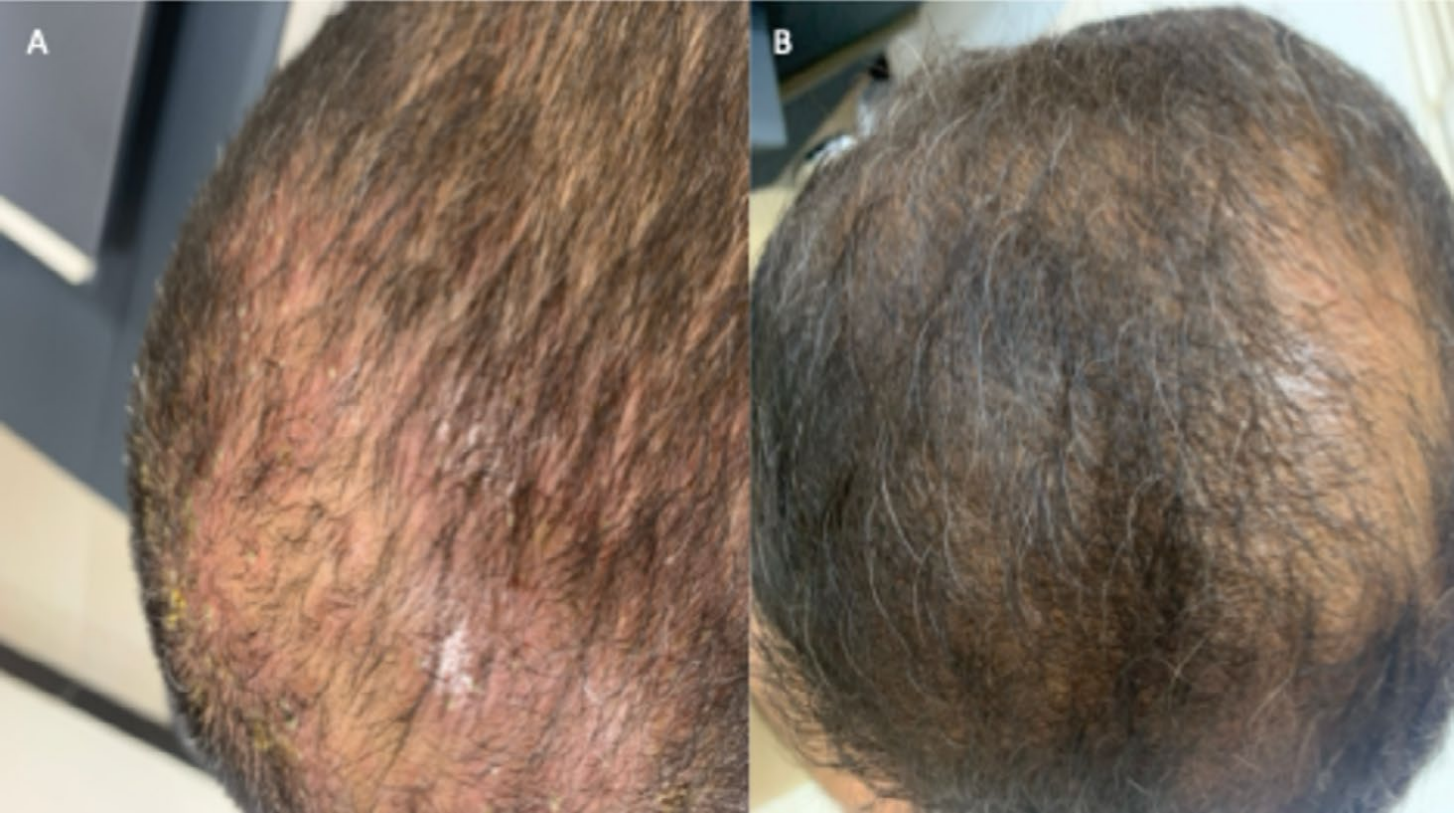
(A) Baseline: Confluent erythematous plaques with thick greasy scale over the vertex and frontal scalp. (B) Six months after final session: Complete clearance with restoration of healthy scalp skin.

The patient underwent three sessions of a 1726 nm laser (Accure; Accure Acne Inc., USA, in collaboration with Quanta System, Italy) at 2‐week intervals, targeting sebaceous glands with real‐time epidermal temperature monitoring. Boost mode was used with continuous contact cooling to maintain safe thermal control. The skin was cooled to approximately −1°C, and laser delivery was automatically initiated, delivering six pulses per spot. The peak epidermal temperature (PET) was set at 41°C. Each treatment area was divided into grids, and a stamping technique was applied with three passes per section (vertical lines, horizontal lines, and dots) to ensure uniform coverage. Laser energy and power were software‐controlled, and the total energy per session was determined automatically to ensure reproducible dosing.

Topical anesthetic cream and contact cooling were used for pain management. The procedure was well tolerated, with a VAS pain score of approximately 3/10, and all sessions were completed without interruption or additional analgesia. No topical or systemic adjunctive therapy, including antifungals or corticosteroids, was used during or after the treatment course.

At 6 months after the final session, there was near‐complete resolution of erythema, scaling, and pruritus, with restoration of clinically healthy scalp (Figure 1B). No post‐inflammatory hyperpigmentation, blistering, or other adverse effects were observed, despite the patient's Fitzpatrick skin phototype IV. No maintenance therapy was required during follow‐up, and the patient reported high satisfaction with both symptomatic and cosmetic outcomes.

Visible hair regrowth was noted in treated areas; however, this is interpreted as a secondary effect of resolution of chronic inflammation and scaling, rather than a direct stimulatory effect of the laser on hair follicles.

Seborrheic dermatitis is a chronic inflammatory dermatosis affecting sebaceous gland–rich areas, with a prevalence of 1%–5% in adults, particularly males [1, 2]. Its pathogenesis involves sebaceous hyperactivity, altered immune responses, and Malassezia spp. colonization [3, 4]. Conventional therapies often provide short‐term relief and may be limited by irritation, tachyphylaxis, or cutaneous atrophy [5]. The 1726 nm laser, originally developed for acne vulgaris, selectively targets lipid‐rich sebaceous glands via photothermolysis while sparing the epidermis, representing an upstream therapeutic approach in SD management [6]. This case illustrates a favorable and sustained clinical response of refractory scalp seborrheic dermatitis following a short, predefined 1726 nm laser treatment series in a Fitzpatrick phototype IV patient, in the absence of adjunctive pharmacologic therapy. Limitations include the single‐case design, relatively short follow‐up for a relapsing disease, and the lack of high‐resolution baseline images. Nevertheless, these findings support further controlled studies evaluating sebaceous‐targeted laser therapy in seborrheic dermatitis.

## Funding

No source of funding was received.

## Ethics Statement

The authors have nothing to report.

## Consent

Written informed consent was obtained from the patient's legal guardians for the publication of this case report, including clinical details and imaging findings while ensuring confidentiality and anonymity.

## Conflicts of Interest

The authors declare no conflicts of interest.

## Data Availability

The data that support the findings of this study are available from the corresponding author upon reasonable request.
